# Supplementary material for: Electrophysiological resting-state signatures link polygenic scores to general intelligence
Source: Sci Rep. 2025 Nov 21;15:41170. doi: 10.1038/s41598-025-26778-4 (PMC12638761; doi:10.1038/s41598-025-26778-4)
Supplement: Supplementary file 1 — Supplementary Material 1 [file 41598_2025_26778_MOESM1_ESM.pdf]

Supplementary Material

Electrophysiological resting-state signatures link polygenic scores to general intelligence

Rebecca Engler[a], Christina Stammen[a], Stefan Arnau[b], Javier Schneider Penate[c], Dorothea Metzen[d], Jan Digutsch[a,e], Patrick D. Gajewski[b], Stephan Getzmann[b], Christoph Fraenz[a], Jörg Reinders[f], Manuel C. Voelkle[g], Fabian Streit[h,i,j], Sebastian Ocklenburg[k,l,m], Daniel Schneider[b], Michael Burke[a], Jan G. Hengstler[f], Carsten Watzl[n], Michael A. Nitsche[a,o,p], Robert Kumsta[p,q,r], Edmund Wascher[b,p], & Erhan Genç[a*]

[a]: Department of Psychology and Neurosciences, Leibniz Research Centre for Working Environment and Human Factors at the Technical University of Dortmund (IfADo), 44139 Dortmund, Germany
[b]: Department of Ergonomics, Leibniz Research Centre for Working Environment and Human Factors at the Technical University of Dortmund (IfADo), 44139 Dortmund, Germany
[c]: Department of Neuropsychology, Institute of Cognitive Neuroscience, Faculty of Psychology, Ruhr University Bochum, 44801 Bochum, Germany
[d]: Institute of Psychology, Department of Educational Sciences and Psychology, TU Dortmund University, 44227 Dortmund, Germany
[e]: Institute of Behavioral Science and Technology, University of St. Gallen, 9000 St. Gallen, Switzerland
[f]: Department of Toxicology, Leibniz Research Centre for Working Environment and Human Factors at the Technical University of Dortmund (IfADo), 44139 Dortmund, Germany
[g]: Department of Psychology, Humboldt-Universität zu Berlin, 10117 Berlin, Germany
[h]: Department Genetic Epidemiology in Psychiatry, Central Institute of Mental Health, Medical Faculty Mannheim, Heidelberg University, 68159 Mannheim, Germany
[i]: Department of Psychiatry and Psychotherapy, Central Institute of Mental Health, Medical Faculty Mannheim, Heidelberg University, 68159 Mannheim, Germany
[j]: Hector Institute for Artificial Intelligence in Psychiatry, Central Institute of Mental Health, Medical Faculty Mannheim, Heidelberg University, 68159 Mannheim, Germany
[k]: Department of Psychology, Medical School Hamburg, 20457 Hamburg, Germany
[l]: ICAN Institute for Cognitive and Affective Neuroscience, Medical School Hamburg, 20457 Hamburg, Germany
[m]: Biopsychology, Institute of Cognitive Neuroscience, Faculty of Psychology, Ruhr University Bochum, 44801 Bochum, Germany
[n]: Department of Immunology, Leibniz Research Centre for Working Environment and Human Factors at the Technical University of Dortmund (IfADo), 44139 Dortmund, Germany
[o]: Bielefeld University, University Hospital OWL, Protestant Hospital of Bethel Foundation, University Clinic of Psychiatry and Psychotherapy, 33615 Bielefeld, Germany
[p]: German Center for Mental Health (DZPG), partner site Bochum/Marburg, Germany
[q]: Genetic Psychology, Faculty of Psychology, Ruhr University Bochum, 44801 Bochum, Germany
[r]: Department of Behavioural and Cognitive Sciences, Laboratory for Stress and Gene-Environment Interplay, University of Luxemburg, 4366 Esch, Luxemburg

*Corresponding author and lead contact: genc@ifado.de

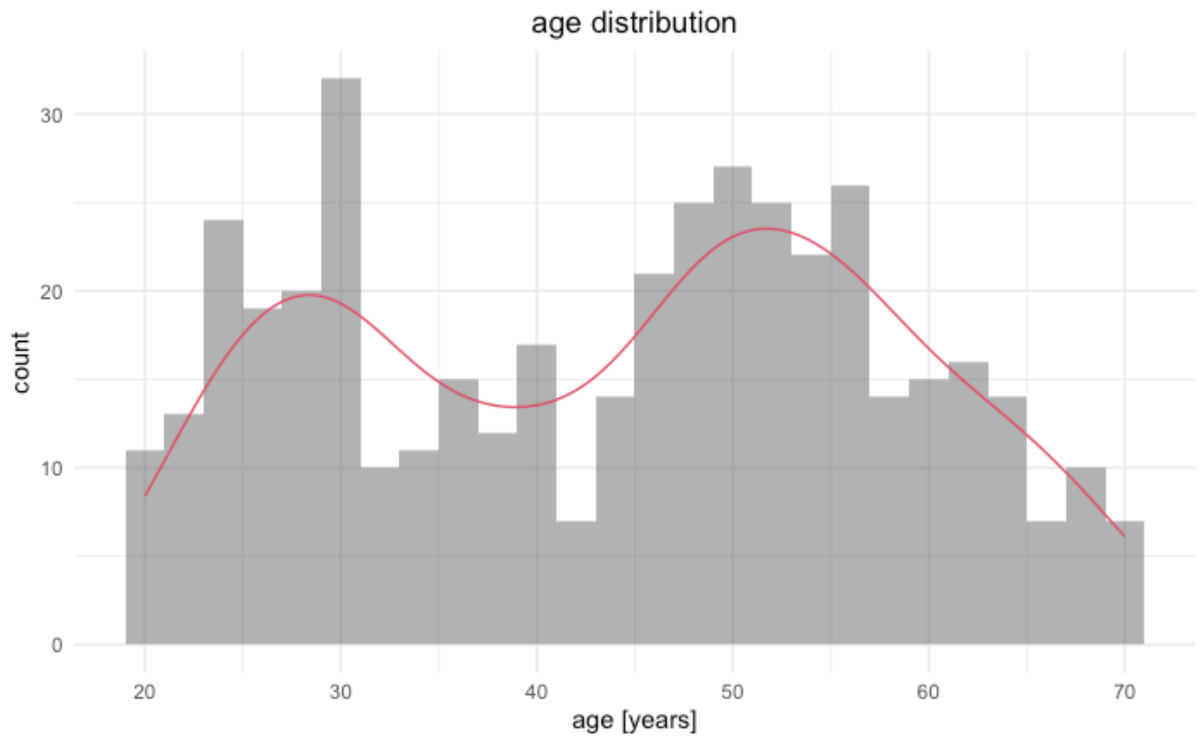

Supplementary Figure S1. Age distribution in the whole sample.

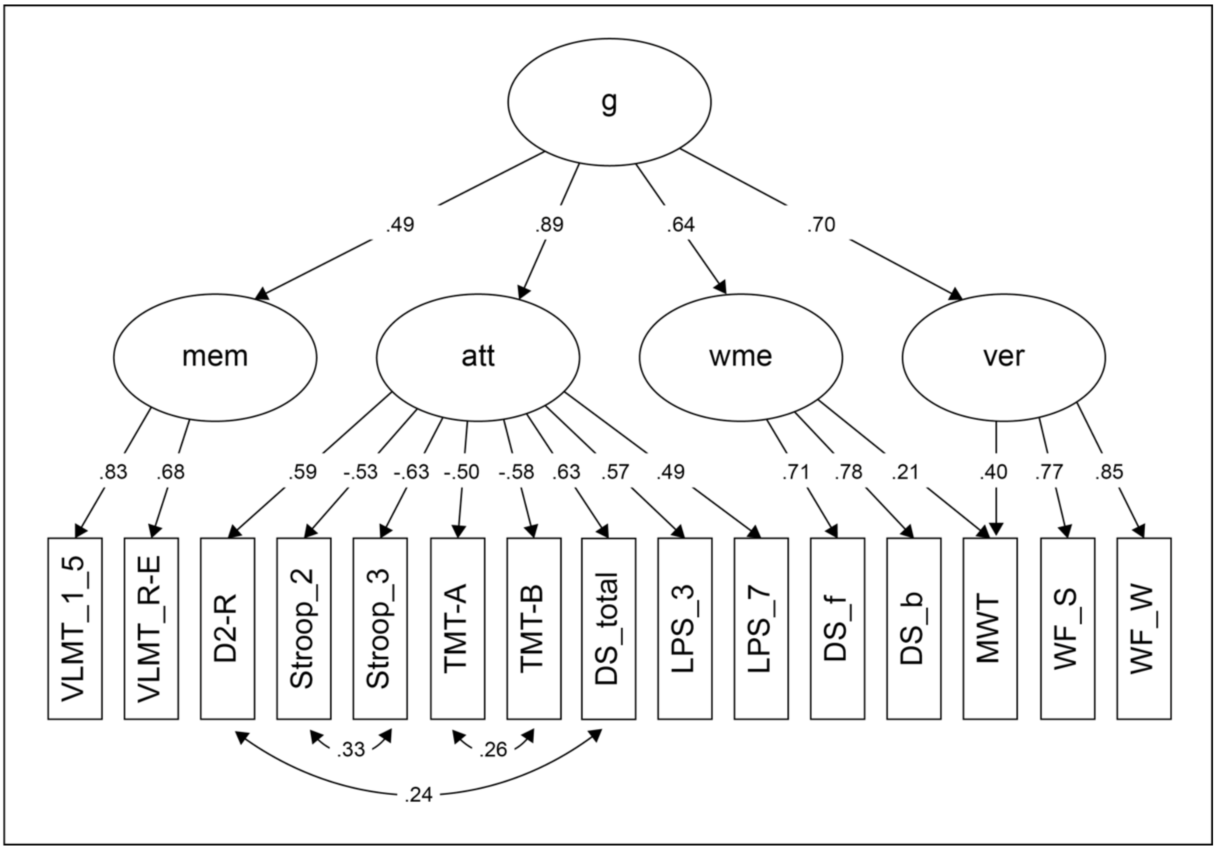

Supplementary Figure S2. Confirmatory factor analytic model. *g* = general factor of intelligence, mem = verbal memory, att = attention, wme = working memory, ver = verbal fluency. VLMT = Verbal Learning and Memory Task, TMT = Trail Making Test, DS = Digit Span, LPS = Leistungsprüfsystem, MWT = Multiple Choice Vocabulary Test, WF = Word Fluency.

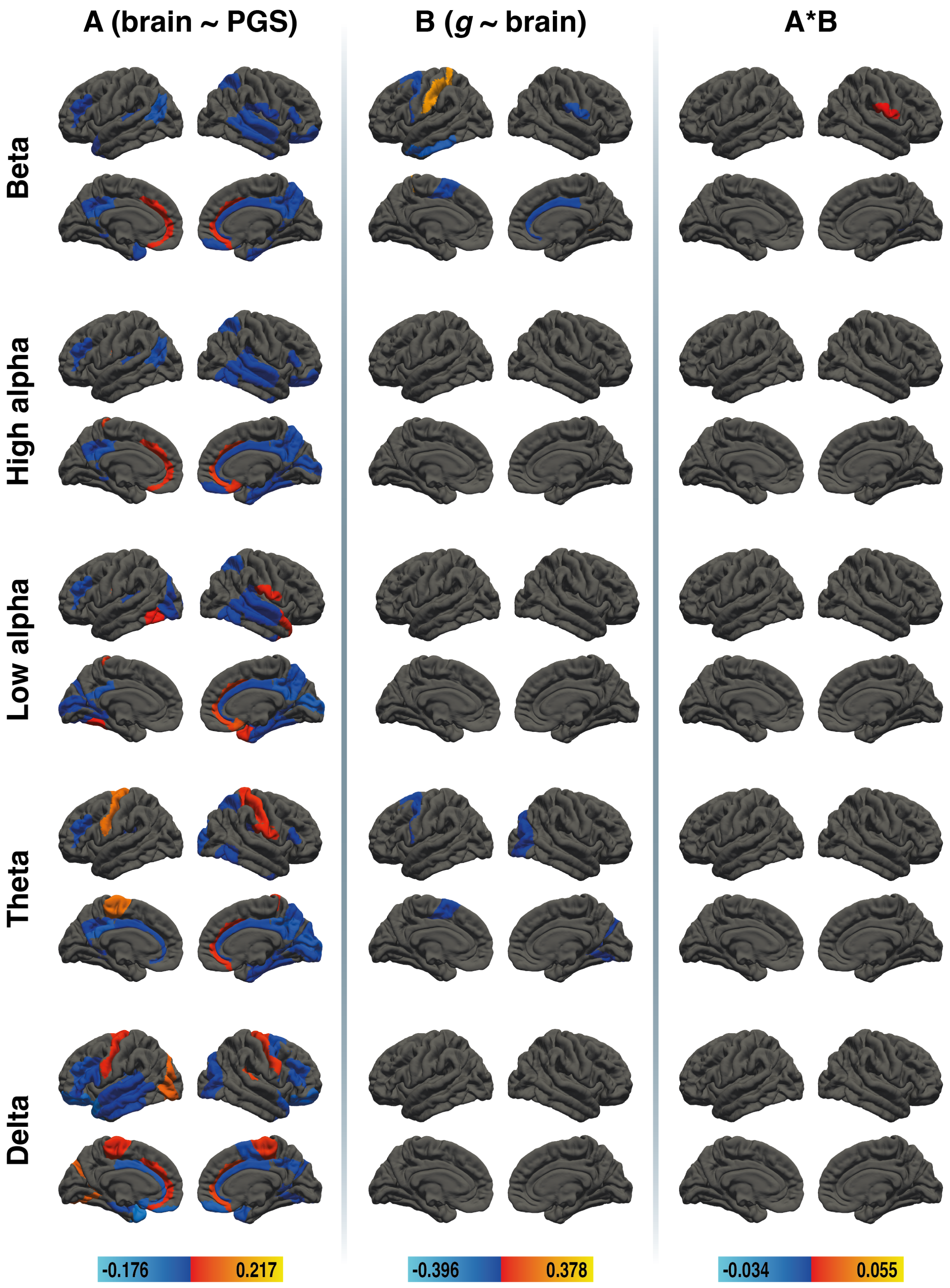

Supplementary Figure S3. Results of the region-specific mediation analysis via elastic-net regression (nodal efficiency). Respective mediators were 41 cortical areas in the left and 41 cortical areas in the right hemisphere of five different frequency bands (delta, theta, low alpha, high alpha, and beta, from the bottom to the top). The figure shows the results from path a analysis (brain ~ PGS), path b analysis (g ~ brain), and the mediation effect (from left to right). Brain surfaces are shown in lateral and sagittal view, for the left and right

hemisphere. Positive effects are depicted in red and yellow, negative effects are depicted in blue. The figure was created with Adobe Illustrator 2020 (Adobe Inc., San Jose, CA, USA).

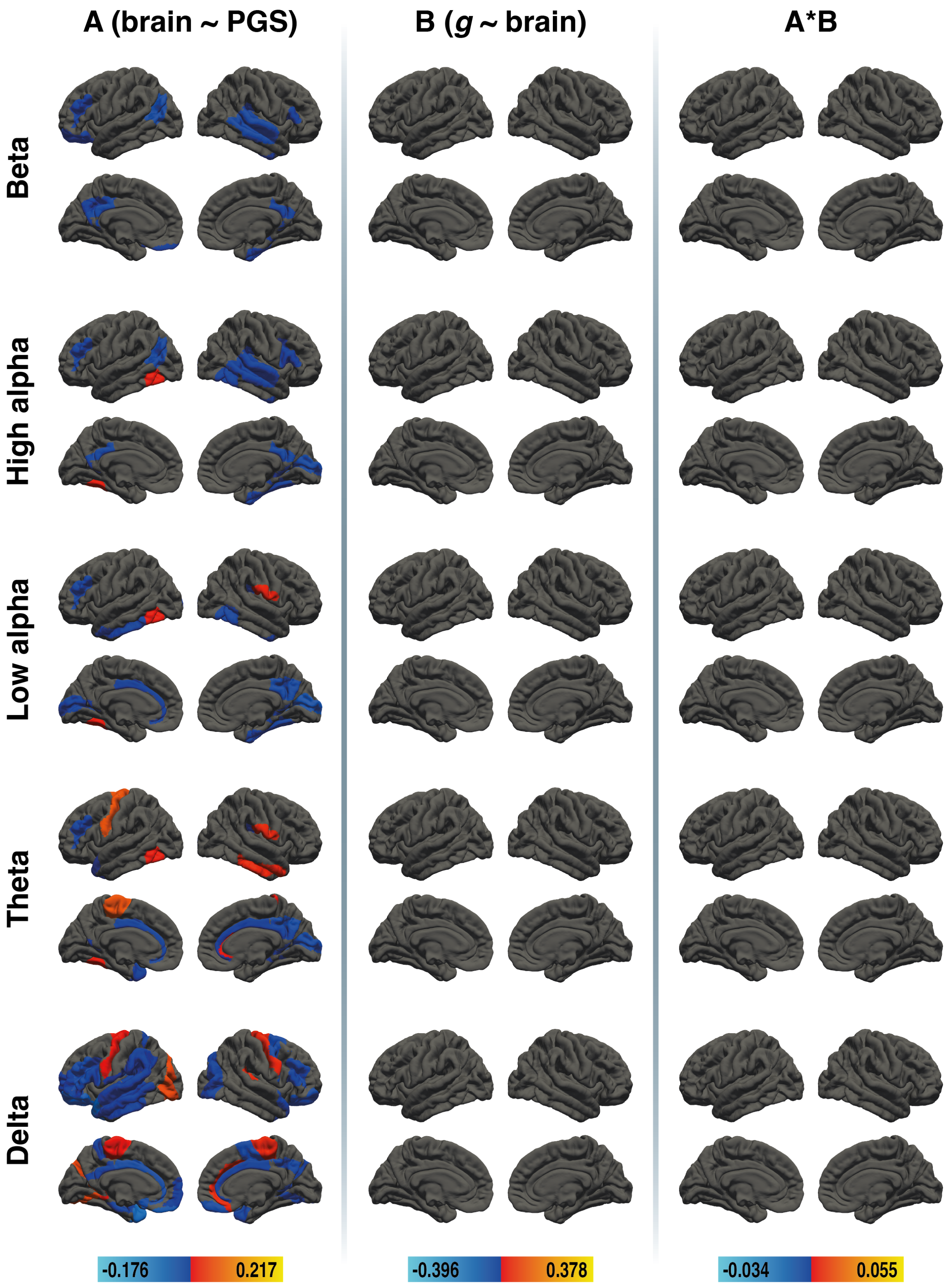

Supplementary Figure S4. Results of the region-specific mediation analysis via elastic-net regression (local clustering). Respective mediators were 41 cortical areas in the left and 41 cortical areas in the right hemisphere of five different frequency bands (delta, theta, low alpha, high alpha, and beta, from the bottom to the top). The figure shows the results from path a analysis (brain ~ PGS), path b analysis (g ~ brain), and the mediation effect (from left to right). Brain surfaces are shown in lateral and sagittal view, for the left and right hemisphere. Positive effects are depicted in red and yellow, negative effects are depicted in blue. The figure was created with Adobe Illustrator 2020 (Adobe Inc., San Jose, CA, USA).

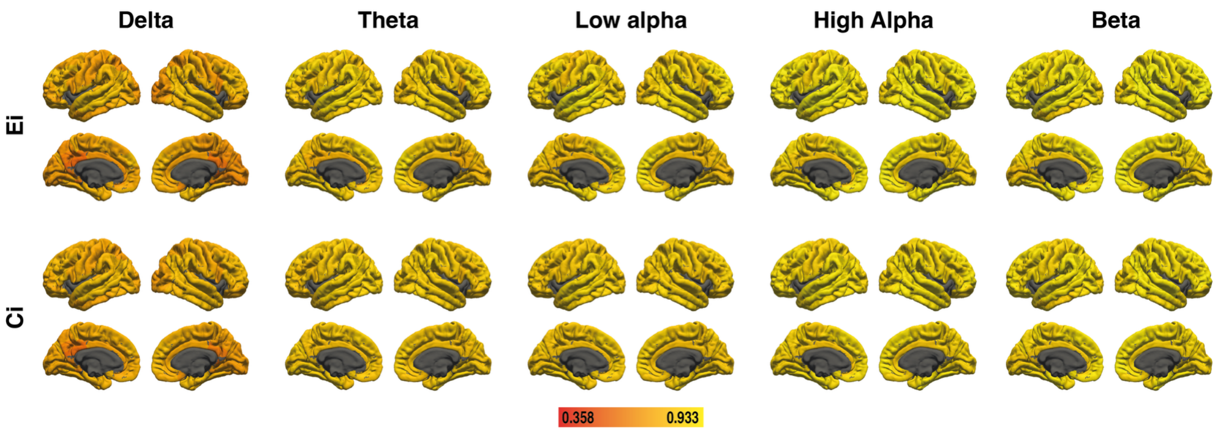

Supplementary Figure S5. Test-retest reliability of nodal efficiency (E_i) and local clustering (C_i) as measured by ICC. Lighter colors indicate higher reliability. The figure was created with Adobe Illustrator 2020 (Adobe Inc., San Jose, CA, USA).

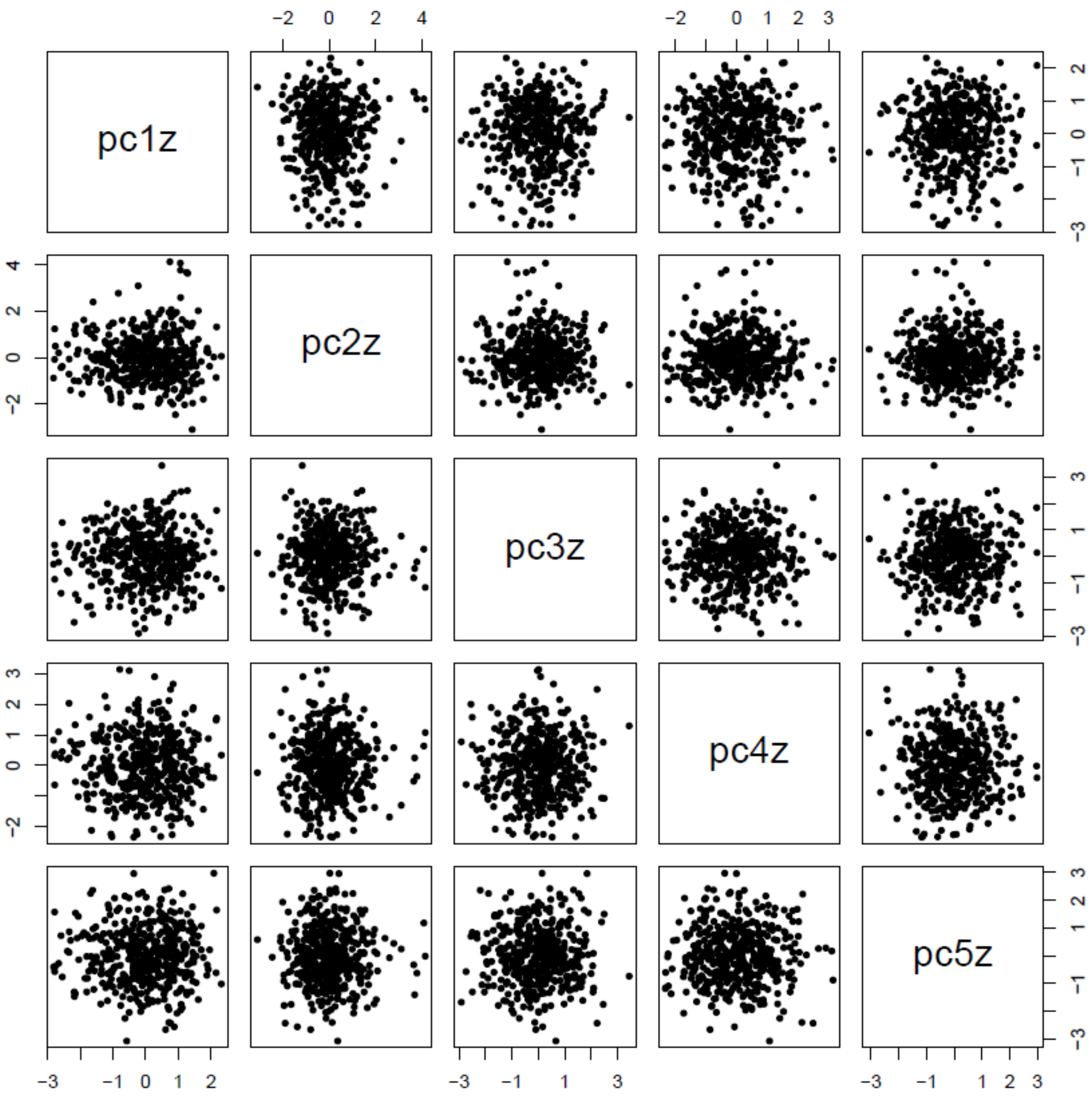

Supplementary Figure S6. Scatter plots of the first five genetic principal components. Each panel displays a scatter plot of a pair of the first five genetic principal components extracted from preprocessed genotyped data. The location of each data point is given by an individual's z-scored projections of two paired components. Genetic homogeneity of the sample is indicated by the data points gravitating towards the center of each panel. Pcz, principal components (z-scored).

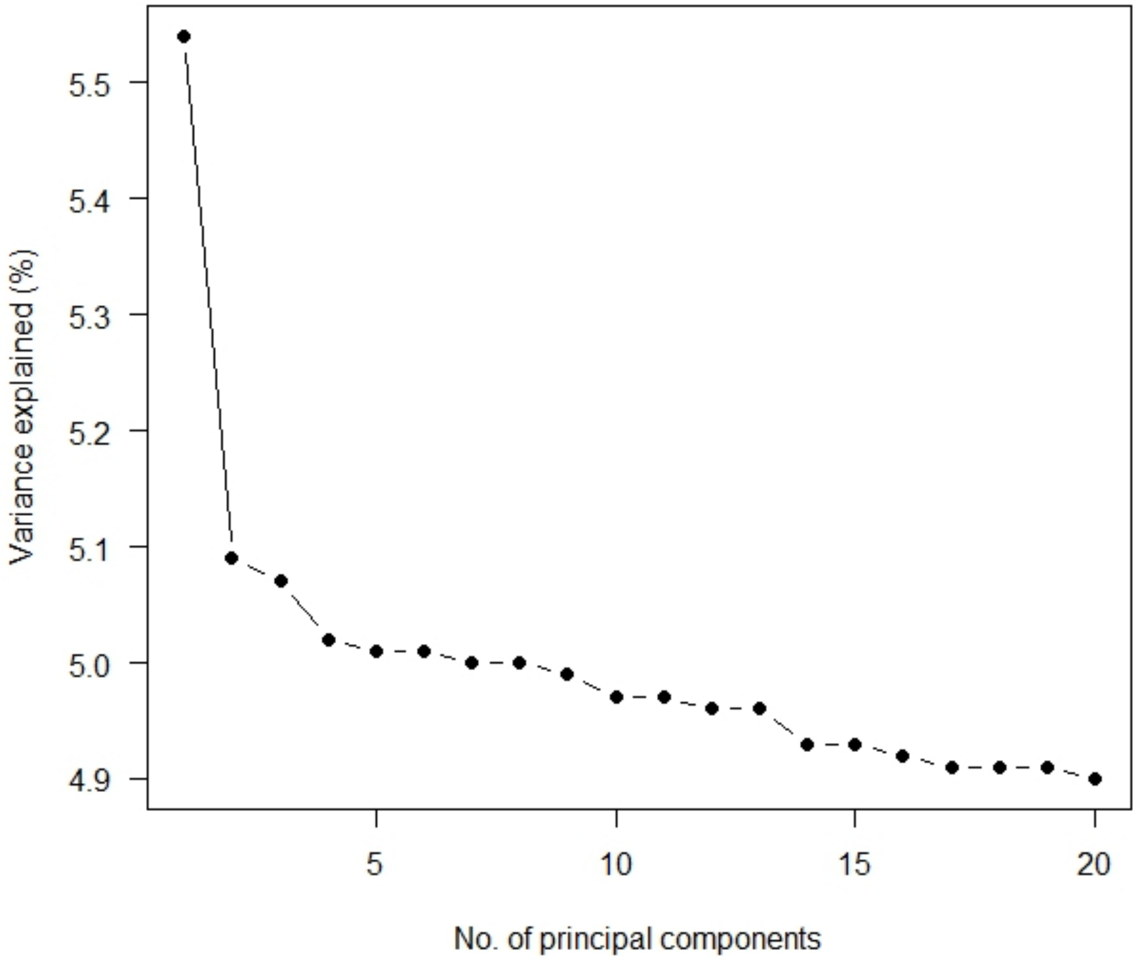

Supplementary Figure S7. Scree plot of genetic principal components. Principal components were extracted from the preprocessed genotyped data. The variance explained (y-axis) drops with each additional principal component (x-axis). A steep drop after the first component, followed by a second modest drop after the third component before the slope levels off suggest that the first two to four components sufficiently capture the variability in genetic ancestry.

Acknowledgments

The authors are grateful to Tobias Blanke for technical support and to the lab staff, in particular to Claudia Brockhaus, Pia Deltenre, Barbara Foschi, Carola Reiffen, Silke Joiko, Christiane Westedt and their team of student assistants for their help with data acquisition. The study is endorsed by the German Center for Mental Health (DZPG).
